# A Patient With Pelizaeus-Merzbacher Disease Caused by a c.67G>A Mutation in the PLP1 Gene

**DOI:** 10.7759/cureus.42458

**Published:** 2023-07-25

**Authors:** Mohammad Usman, Alexis Koch, Laurence Stolzenberg, Austin Huang, Veronica E Nkie, Mohammad Ibrahim

**Affiliations:** 1 Psychiatry and Behavioral Sciences, Alabama College of Osteopathic Medicine, Dothan, USA; 2 Internal Medicine, Alabama College of Osteopathic Medicine, Dothan, USA; 3 Orthopaedic Surgery, Alabama College of Osteopathic Medicine, Dothan, USA; 4 Neurology, Alabama College of Osteopathic Medicine, Dothan, USA; 5 General Surgery, Alabama College of Osteopathic Medicine, Dothan, USA; 6 Health Sciences, University of Central Florida, Orlando, USA

**Keywords:** pediatric intensive care unit (picu), neonatal intensive care unit (nicu), nicu, pediatric genetics, genetics

## Abstract

Pelizaeus-Merzbacher disease is a tremendously rare genetic disorder caused by a mutation on the X chromosome. The mutation affects a gene critical to white matter myelination and results in significant neurological issues. Here, we present one such case of a child diagnosed with Pelizaeus-Merzbacher disease. A relatively normal gestation and birth belied the underlying issue until he presented to the emergency department a month after birth with seizure-like activity and failure to thrive. After intensive evaluation and treatment, the patient was diagnosed with the illness and received surgery to place a tracheostomy and a gastrostomy tube to treat the stridor and failure to thrive caused by his illness. After approximately a month and a half of inpatient treatment, the patient was able to be discharged home in stable condition.

## Introduction

Pelizaeus-Merzbacher disease is an extremely rare genetic disorder that results in serious neurological issues including ataxia, developmental delay, and intellectual disability [1,2]. The disease is a type of leukodystrophy, and it can cause significant abnormalities in the white matter of both the brain and the spinal cord. The incidence of Pelizaeus-Merzbacher disease is very low, with some estimates of the prevalence ranging between one in 200,000 male births and one in 500,000 [3,4]. The genetics of typical Pelizaeus-Merzbacher disease is that of an X-linked illness, and the gene most conclusively linked to the disease is the proteolipid protein 1 (*PLP1*) gene, located on the long arm of the X chromosome [5]. This gene is one that is partially responsible for the myelination of white matter via the production of a protein critical to the process of myelination called the myelin proteolipid protein. The specific underlying pathophysiological cause of the illness varies from case to case, but it is most commonly because of a duplication of the region on the X chromosome where the *PLP1* gene is located. In addition, missense mutations have been known to cause the disease as well and are commonly seen in patients with the connatal form of the illness [6,7].

There is a wide range of possible phenotypes exhibited by patients afflicted with the disease, ranging from mild to severe. There are generally considered to be two types of illness: classical and connatal. The classical form of the illness is the more common type, which typically presents with the aforementioned neurological issues several years into life. While they continue to develop at a reduced rate until adolescence, eventually, most patients begin to experience developmental regression and worsening motor control [4,6]. The connatal form of the illness is much more severe and typically presents early in infancy. These patients often have issues with feeding, breathing, and speech in particular [4,6,7]. While patients with the connatal form also experience developmental delay, they have much more severe symptoms. Most are unable to walk or speak, although they may be able to understand speech [4].

There are currently no therapies that can treat the underlying genetic issue, so treatment is generally based on managing symptoms. Patients with milder forms of the disease may have normal life spans, but those with severe expressions of illness often have greatly reduced life spans [2,3]. In addition to the reduced life span, patients will often face debilitating side effects that greatly reduce quality of life that often require significant medical intervention. There is ongoing research on potential genetic therapies that have shown promise in controlling symptoms in animal models, but further research is needed to provide some relief to those suffering from this illness [8].

## Case presentation

The patient was born at 39.5 weeks as a spontaneous vaginal delivery and initially appeared normal, with an appearance, pulse, grimace, activity, and respiration (APGAR) score of 8 at one minute and 9 at five minutes. In addition, a slight pectus excavatum was noted. However, 15 minutes after delivery, the patient began to experience significant respiratory distress, stridor, and desaturation. As a result, the patient was transferred to the neonatal intensive care unit (NICU) for intervention. After being placed on a continuous positive air pressure (CPAP) machine, the patient’s condition improved. Further investigation showed laryngomalacia and intermittent paradoxical vocal cord movement, which was believed by the consulted ear, nose, and throat (ENT) physician would resolve on its own. After slowly being weaned off the CPAP and given supportive care for a week, the patient was discharged home with the mother with an expected outpatient follow-up.

The patient presented again to the emergency room (ER) approximately one month after birth for new-onset seizure activity. The mother reported that the patient was experiencing rapid eye movements and side-to-side head movements. Each episode was around 5-10 seconds long, after which the patient would return to normal. On presentation to the ER, the patient appeared in no distress and was afebrile and hemodynamically stable, although the patient’s mother reported that the patient tended to breathe harshly. He also had not been gaining enough weight at home, despite normal feedings. It was noted that the patient’s growth percentile had fallen from the 59th percentile at birth to the 23rd percentile upon presentation and was therefore diagnosed with failure to thrive. The patient was admitted to the hospital for further workup and evaluation.

Vital signs on admission showed normal oxygen saturation on room air with normal blood pressure. There was no tachycardia or tachypnea noted. A comprehensive metabolic panel (CMP) and a urinalysis (UA) were performed, and all values were within normal limits. A chest X-ray (CXR) was performed, which did not show any pathologies. An electroencephalograph (EEG) showed no abnormalities, but the physical examination showed significantly abnormal eye movements. A magnetic resonance imaging (MRI) of the brain was performed but did not show any masses, lesions, plaques, or any other pathologies. Supportive care was planned upon admission to the epilepsy management unit. Thorough family history revealed that the mother had several brothers who died in childhood due to an unspecified neurological issue. She reported that her brothers all failed to thrive and had significant respiratory issues and serious issues with ambulation, as well as speech pathologies. The mother was an immigrant who grew up in a developing country, and as such, her brothers were never tested to determine what the underlying cause of their symptoms was. Because of this history, genetics was then consulted. Cerebral creatine deficiency syndrome was initially suspected, and a full genomic analysis was ordered. Genomic analysis revealed a hemizygous c.67G>A (pGly23Arg) point mutation in the *PLP1* gene, definitively diagnosing the patient with Pelizaeus-Merzbacher disease. Analysis of the mother’s and father’s genomes revealed that the mother was heterozygous for the mutation, while the father was negative for any mutation.

**Figure 1:**
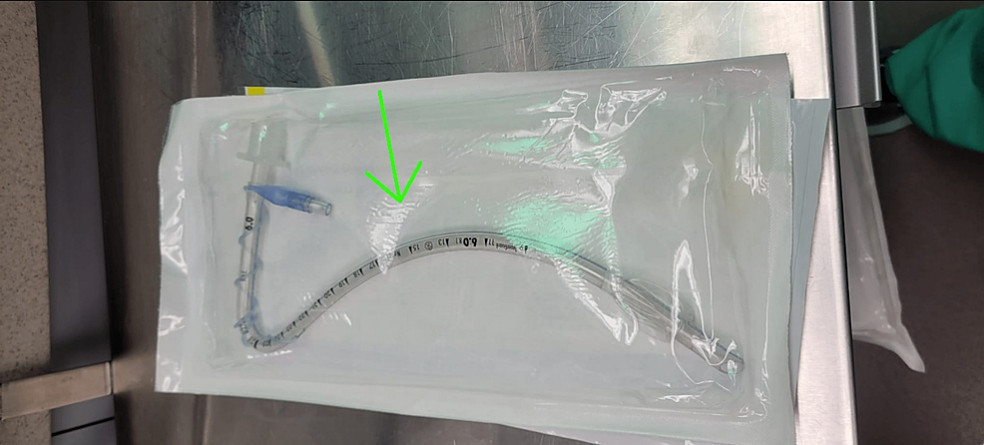
Pediatric tracheostomy tube

Several days after admission, the patient began to experience significant stridor. ENT was consulted, and a bronchoscopy was performed, which revealed paradoxical vocal cord movement with inappropriate closure. A swallow study revealed penetration to the vocal cords. A gastrointestinal (GI) physician was consulted as well, who advised that a gastrostomy tube would be necessary to ensure proper weight gain. A nasogastric (NG) tube was placed in the interim to ensure proper feeding until surgery could be performed. It was also decided that a tracheostomy (Figure 1) would be performed, and 12 days after the initial presentation to the hospital, the patient underwent surgery to have the tracheostomy and gastrostomy tube placed. The operation proceeded as planned with no complications, and the patient was discharged to the pediatric intensive care unit (PICU). The patient’s condition continued to improve, and approximately a month and a half after the initial presentation to the emergency department, the patient was discharged home with instructions to closely follow-up with his pediatrician.

## Discussion

This patient posed an interesting conundrum to diagnose during the initial presentation to the emergency department. While his post-birth clinical course was serious and required a prolonged stay in the hospital to treat his laryngomalacia and vocal cord spasm, there was nothing that indicated that there might have been grave underlying pathology that could seriously impair normal development. The initial presenting symptoms of eye and head movement would direct any clinician to consider a seizure disorder at first glance, but the normal laboratory results and imaging belied that diagnosis [4]. This case is an excellent example of the potential benefits of genetic analysis for the physician’s ability to diagnose and treat unusual presentations [2]. While the chance of detecting such an atypical disease in the average patient is virtually zero, it should always be considered as an option by the thoughtful clinician when the initial workup does not reveal the underlying diagnosis.

In addition, his severe neurological issues typical with the connatal form of Pelizaeus-Merzbacher disease pose a grave issue to quality of life and health [1,5]. It is therefore critically important for the clinician to be able to ensure that the patient will be able to get adequate care at home, and as such, educating caregivers on the proper usage of the tracheostomy and gastrostomy tubes that the patient has is critical to safeguard the patient from any complications. In this case, the multidisciplinary team was able to educate the patient’s parents appropriately and sufficiently on the best ways to manage his interventions, and they continue to excellently manage his home care. Even though a lack of effective treatment options for such a rare case poses great difficulty in the management of care, it is vitally important that the patient’s caregivers be given all the information about the prognosis, even when that prognosis is poor [4,8]. While the patient is likely to suffer developmental delay and continually worsening neurological symptoms, with constant monitoring and careful consideration of supportive treatment options, the clinicians in charge of his treatment continue to ensure that he is taken care of so that he may have the best quality of life he can.

## Conclusions

We hope that this case can clue clinicians into considering Pelizaeus-Merzbacher disease when presented with a young patient presenting with serious neurological issues. While obviously, the rarity of the illness means that it should not be at the top of the differential when evaluating patients, it certainly merits further testing and investigation. In particular, genetic testing can be very useful in elucidating underlying chromosomal abnormalities and possibly explaining the presenting symptoms. In addition, the knowledge of the fundamental pathophysiological cause of this patient’s illness can be used to guide treatment options to best manage his care.
